# Smoking knowledge and decision in an era of widespread awareness: Persistent disparities and policy implications

**DOI:** 10.1371/journal.pone.0329691

**Published:** 2025-08-04

**Authors:** Miguel Antonio Garcia Estrada, Rutcher Madera Lacaza, Kent Jason Go Cheng

**Affiliations:** 1 Department of Public Administration and Policy, School of Public and International Affairs, University of Georgia, Athens, GeorgiaUnited States of America; 2 School of Statistics, University of the Philippines, Quezon City, Metro Manila, Philippines; 3 Congressional Policy and Budget Research Department, House of Representatives, Quezon City, Metro Manila, Philippines; 4 Center for Healthy Aging, College of Health and Human Development, The Pennsylvania State University, University Park, Pennsylvania, United States of America; National Institutes of Health, University of the Philippines Manila / De La Salle University, PHILIPPINES

## Abstract

Years of anti-smoking campaigns to promote public awareness about smoking’s negative health effects have resulted in increased awareness across a large segment of the population. Amid gains in smoking-related knowledge, important but unexplored questions remain about population-level trends and the continuing importance of knowledge-based anti-smoking policies. Using the Philippine case, this paper is the first to examine persistent disparities in knowledge on smoking’s health risks and the continued importance of knowledge-based interventions in discouraging smoking. We also investigated whether knowledge moderates the effect of price on smoking decisions. For this cross-sectional study, we conducted ANOVA, Tukey HSD tests, and a two-part multivariate regression model on the 2009, 2015, and 2021 waves of the Philippine Global Adult Tobacco Survey. We used daily cigarette sticks smoked as the dependent variable and (a) a binary and discrete variable measuring smoking health risk, (b) a categorical variable for wealth quintile, and (c) an interaction term as the main independent variables. We found that knowledge on smoking’s ill health effects, by itself, continues to be negatively associated with smoking, even as 9 out of 10 individuals already possess knowledge about smoking’s health risks. Individuals who view smoking as addictive and as having ill health effects smoke 1 stick (10.8% of average sticks) less. Persistent disparities in knowledge between the lowest and highest wealth groups were observed. Despite significant gains in population-wide smoking-related knowledge, disparities across socioeconomic groups persist. Closing knowledge-based disparities calls for approaches that are tailored to the needs of different groups, particularly less affluent individuals.

## Introduction

Policies increasing the public’s knowledge about smoking-related health risks are integral to addressing the smoking epidemic. The World Health Organization (WHO) Framework Convention on Tobacco Control (FCTC) underscores the importance of adopting non-price-based policies, in tandem with price-based policies, to discourage smoking and reduce smoking prevalence. Evidence exists on the importance of non-price-based measures, such as health warnings and tobacco advertisement bans, just as evidence supports the role of increasing tobacco prices through policies like excise tax hikes [1,2].

Due to sustained efforts to increase the public’s awareness, significantly large sections of the population have gained knowledge about the risks involved in smoking. In the Philippines, more than 9 in 10 adults in 2021 knew that smoking is addictive and causes ill health [3]. However, disparities in knowledge about specific smoking-related health risks persist across socioeconomic groups, with individuals in the lowest wealth groups lagging behind those in the highest wealth groups [3]. Despite wide awareness, 1 in 5 adult Filipinos were smokers [4]. Among countries in Southeast Asia, the Philippines has the third highest smoking prevalence [5].

To curb smoking, the Philippine government enacted laws introducing non-price based anti-smoking policies, in addition to tobacco excise taxes. Laws mandating health warnings form an important pillar of increasing the public’s awareness about the deleterious effects of smoking. Two laws provide the basis for relevant government policies in the Philippines: (a) Republic Act no. (RA) 9211 or the Tobacco Regulation Act of 2003 and (b) RA 10643 or the Graphic Health Warnings Law, enacted in 2015 [6,7]. RA 9211 provided for the inclusion of warnings about the health effects of direct and secondhand smoking in designated smoking areas. The law also banned tobacco advertisement. Moreover, RA 9211 mandated the inclusion of text warnings in cigarette packs, with messages ranging from “Cigarette Smoking is Dangerous to your Health” to “Smoking Kills.”

RA 10643 strengthened the earlier policy by mandating graphic warnings, in addition to text warnings, showing images of smoking-related diseases [7]. The law specified that graphic and text warnings be printed on 50% of the front and back portions of each cigarette pack. Enacting the policy aligns with Article 11 of the Framework Convention on Tobacco Control [1]. In this study, in referring to anti-smoking campaigns, we emphasize non-price based interventions aimed at reducing smoking prevalence while also encouraging cessation, such as health warnings, advertising bans, and public education efforts [8,9]. These measures are consistent with the objectives of Articles 11–13 of the WHO FCTC [10].

On price-based interventions, two excise tax reforms enacted in the past decade made cigarettes drastically more expensive. The first is the Republic Act no. 10351, also known as the Sin Tax Reform Act of 2012. A US$0.09 pack of cigarettes was taxed US$0.05 pre-reform but when the law took effect in 2013, the same pack was taxed US$0.24 or nearly three times as much. In addition, the 2012 reform shifted the tax system from multi-tier (i.e., taxes then depended on tiers set according to net retail price per pack wherein the higher the net retail price per pack, the higher the tax rate assigned) to unitary starting 2017, simplifying the tax regime. In a subsequent reform, Republic Act 11346 which was passed 2019, raised the unitary cigarette excise tax further and set a 5% increase per annum starting 2023 [11–13]. A cost-effectiveness study found that the 29% increase in cigarette price brought about by RA 11346 saved US$10,600–11,950 per tobacco-related disability-adjusted life years averted [12,14].

Risk perceptions may play a role in explaining the relationship between smoking and price. While smoking risk perception is a multi-faceted concept, the measure we are interested in takes into account one’s knowledge of smoking-induced diseases [15]. Defining risk perception in this sense, we posit that both price and risk perceptions can act as signals about the deleterious health impacts of smoking, with risk perceptions intensifying the effect of price [16]. Uncovering the role of risk perceptions may illuminate why price is negatively related to smoking participation but not smoking intensity. After all, little is known about the relationship between the two factors.

Consistent with the literature, we posit that better appreciation of smoking risks, measured in terms of knowledge about the ill health effects of smoking, is negatively associated with the decision to smoke [17–19]. Moreover, we hypothesize that risk perception moderates the relationship between price and the decision to smoke. Specifically, individuals more knowledgeable about the deleterious effects of smoking are more sensitive to price changes because like price, knowledge increases the cost of smoking and decreases the satisfaction derived from smoking [20]. Moreover, we recognize that differences in risk perceptions are rooted in underlying socioeconomic factors. Following the fundamental cause theory (FCT) [19], we consider resources which broadly include “money, knowledge, power, prestige, and the kinds of interpersonal resources embodied in the concepts of social support and social network” to be important for health.

In this cross-sectional study, we examined whether knowledge on the ill health effects of smoking is negatively related to the number of sticks smoked. This question was considered against the backdrop of significant gains in knowledge on smoking-related health risks over the years, with almost every individual now possessing knowledge on the harms of smoking. Moreover, we investigated whether knowledge acts as a moderator in the relationship between cigarette price and smoking. An underlying issue that was addressed is whether disparities in knowledge on smoking-related health risks persist between individuals in lowest and highest wealth groups. In measuring knowledge about smoking’s ill health effects, we adopted the idea of risk perceptions, conceptualized through three main elements: (a) multiple smoking-related health harms for a (b) definite timeframe among (c) individuals directly involved in cigarette use [15].

## Methods

### Data

The 2009, 2015, and 2021 waves of the Philippine Global Adult Tobacco Survey (GATS) were used for this cross-sectional study. GATS is a nationally representative cross-sectional survey periodically implemented to track tobacco use among adults and gather key tobacco control indicators. Data were accessed through the U.S. Centers for Disease Control and Prevention website [21]. Since data used for this study were de-identified, the study was deemed exempt from ethics review in accordance with U.S.federal guidelines.

We pooled the 2009, 2015, and 2021 waves of the GATS. These three waves are the only GATS surveys conducted in the Philippines to date, providing the most comprehensive data available for studying smoking in the country. Pooling the three waves allows the study of trends in smoking behavior over a crucial period coinciding with the implementation of major Philippine tobacco excise tax reform laws beginning 2013. Data from 2009 provide information in the pre-reform period while those from 2015 and 2021 provide information for the post-reform period. After conducting listwise deletion, we ended up with a study sample of 37,500 [N_2021_ = 17,260 (46.0%), N_2015_ = 11,143 (29.7%), N_2009_ = 9,097 (24.3%)].

The dependent variable is smoking intensity, measured in terms of number of cigarette sticks smoked per day. Non-smokers were assigned zero daily sticks smoked. The main independent variables were (a) cigarette price and (b) smoking risk perception, measured in terms of knowledge about smoking-related diseases (i.e., stroke, heart attack, lung cancer). Smokers were asked to identify the price of the cigarettes they purchased (in Philippine peso or PhP), which implies that non-smokers do not have any price to report. However, it would be incorrect to assume that non-smokers do not face cigarette prices since they can still purchase cigarettes if they choose. To address this issue, we assigned the respondents either the average price per stick of their primary sampling unit (PSU) or their type of residence (urban, rural). Doing so also helped with addressing the endogeneity of self-reported price [22].

Knowledge on smoking’s health risks was determined based on respondent’s belief about the health-related risks of smoking. Specifically, we used the number of smoking-induced diseases the respondent knows about (i.e., discrete variable ranging from 0–3). The GATS questionnaire posed the question, “Based on what you know or believe, does smoking tobacco cause the following,” followed by separate items for each smoking-related diseases [3]. In generating the number of smoking-related diseases known to a respondent, we referred to three conditions common in all three waves of the GATS (i.e., stroke, heart attack, lung cancer) and which have been used to construct knowledge scores by earlier research [23].

Following the literature on the determinants of smoking intensity, we controlled for potential confounders including age, sex (1: female, 0: male), wealth quintile, urban residence (1: urban, 0: rural), and year of survey (2009, 2015, 2021) [24,25]. Across country contexts, younger age and being male are consistently associated with higher rates of adopting smoking uptake and intensity [11,19,24]. Lower socioeconomic status (SES)—measured in this study by lower wealth—is also a known risk factor for cigarette use. Individuals with lower SES often face greater barriers to accessing health information and resources, placing them at a disadvantage compared to their more affluent counterparts. While the influence of type of residence varies across contexts, in the Philippines, urban dwellers were more likely to smoke daily and occasionally than their rural counterparts. The higher rates of smoking among urbanites can be attributed to social norms, peer pressure, and greater exposure to advertisements for tobacco products [26].

The pooled dataset spans over a decade marked by several other changes that may influence smoking behavior, such as changes in the policy landscape, expanded access to digital media, and greater exposure to health information during the COVID-19 pandemic [27]. For instance, heightened consumption of health information during the pandemic has been shown to affect health risk perceptions and preventive behavior, including smoking [28–30]. To account for time-specific confounders, we included year fixed effects in all regression models. Doing so allowed us to control for unobserved temporal confounders and isolate the association between smoking outcomes and the main variables of interest [31].

### Statistical analyses

This cross-sectional study involved both descriptive analysis and multivariate regression analysis. For the descriptive analysis, means, standard deviations, and row and column percentages were generated. As a preliminary step, we conducted ANOVA and Tukey HSD post-hoc tests to explore differences in KAP scores across wealth quintiles. This allowed us to assess any underlying wealth-based disparities in knowledge about smoking harms before proceeding with the main analysis. For the regression, we used the two-part model [32], where the decision to smoke (smoking participation) is modelled separately from the number of sticks smoked per day (smoking intensity) before determining the marginal effects of knowledge. Since smoking participation is a binary choice variable, we used a Probit model for the first part (smoking participation). For the second part of the model, we used a generalized linear model (GLM) with gamma family and log link.

All analyses were conducted using Stata 18.0. The **twopm** user-written package [33] was implemented for the estimation of the two-part model. Probit coefficients were converted to predicted probabilities using **margins** command with **dydx** option to facilitate interpretation. Statistical power was determined to be above 80%, following a post-hoc power analysis for minimum detectable effect size. Coefficients for the marginal effect can be interpreted as a unit change of sticks smoked per unit increase in the respondent’s knowledge about specific smoking-related diseases.

## Results

Table 1 provides the descriptive statistics. Based on sample characteristics, almost all individuals (97%) had knowledge that smoking is deleterious to one’s health (Table 1). However, the figure was slightly lower for smokers (95%) than non-smokers (98%). Individuals, in general, knew that stroke, heart attack, lung cancer are smoking-related (average score: 2.7 out of 3). Mean knowledge about smoking-related diseases is slightly lower for smokers (2.58 out of 3) versus non-smokers (2.73 out of 3). The sample had a mean age of 40 years, with slightly more females (51.7% versus 48.3% males), and had more urban dwellers (54.6%).

**Table 1:** Descriptive statistics and two-part model results.

Variables	Descriptive Statistics		
	(1)	(2)	(3)
	Non-smokers (Row Percent)	Smokers (Row Percent)	Total (Column Percent)
Total sample (%)	30,279 (80.7)	7,221 (19.3)	37,500 (100)
Wave			
2021	14,940 (86.6)	2,320 (13.4)	17,260 (46.03)
2015	8,649 (77.6)	2,494 (22.4)	11,143 (29.71)
2009	6,690 (73.5)	2,407 (26.5)	9,097 (24.26)
Sticks smoked (Range: 0–140)	NA	Mean = 9.22	
		SD = 8.42	
Price (Range: PhP 0.05 to 75)	Mean = 3.77	Mean = 3.00	Mean = 3.62
	SD = 2.50	SD = 2.33	SD = 2.50
Knowledge on Tobacco’s Health Effects	Mean = 0.98	Mean = 0.95	Mean = 0.97
	SD = 0.15	SD = 0.21	SD = 0.17
Knowledge x Price			
Knowledge on Diseases Caused by Smoking	Mean = 2.73	Mean = 2.58	Mean = 2.70
	SD = 0.62	SD = 0.78	SD = 0.66
Knowledge x Price			
Age (Range: 15–101)	Mean = 39.89	Mean = 40.34	Mean = 39.98
	SD = 17.15	SD = 14.26	SD = 16.63
Male	11,784 (65.0)	6,343 (35.0)	18,127 (48.3)
Female	18,495 (95.5)	878 (4.5)	19,373 (51.7)
Wealth			
Quintile 1	6,160 (20.3)	2,018 (28.0)	8,178 (21.8)
Quintile 2	5,350 (17.7)	1,537 (21.3)	6,887 (18.4)
Quintile 3	5,726 (18.9)	1,409 (19.5)	7,135 (19.0)
Quintile 4	6,368 (21.0)	1,276 (17.7)	7,644 (20.4)
Quintile 5	6,675 (22.0)	981 (13.6)	7,656 (20.4)
Rural	13,347 (78.4)	3,669 (21.6)	17,016 (45.4)
Urban	16,932 (82.7)	3,552 (17.3)	20,484 (54.6)

Smokers comprised 19.3% of the sample, most of whom were males. Smoking prevalence declined with higher wealth quintiles (28.0% for the lowest wealth quintile vs. 13.6% of the highest quintile). On average, smokers smoked 9 sticks per day, each stick costing PhP 3.00, on average.

More affluent individuals had better knowledge of smoking-related diseases. Across GATS survey waves (2009, 2015, and 2021), individuals in the highest wealth quintile demonstrated a higher mean score for knowledge on smoking-related diseases compared to those in the lowest quintile. We conducted analyses using ANOVA and Tukey HSD post-hoc tests (S1 Table) to examine whether differences in knowledge were significantly different across wealth quintiles. Results showed persistent disparities, with significantly different mean knowledge scores across wealth groups in each year. Notably, we consistently observed disparities in knowledge when comparing the lowest wealth quintile to each of the four higher quintiles.

Wealth-based differences in mean knowledge scores narrowed in the post-2012 excise tax implementation period (2015 and 2021), indicating a convergence in scores between the lowest and highest wealth quintiles that was largely driven by improvements in scores for the lowest quintile (average score from 2.36 in 2012 to 2.75 in 2021). These findings highlight two important facts: (1) the association between socioeconomic status and knowledge about smoking-related harms, and (2) persistent knowledge gaps despite convergence in mean knowledge scores across years.

Table 2 provides the two-part model regression results. The findings, in general, do not lend support to the hypothesis that knowledge moderates the effect of price on smoking (Table 2), given that the interaction term between knowledge and price was not statistically significant. However, knowledge on smoking’s ill health effects, by itself, is negatively associated with an individual’s smoking decision. Individuals who agreed that smoking is addictive and has ill health effects tend to smoke 1 stick (10.8% of average sticks) less, on average (Table 2, column 3). This result is statistically significant at the 1% level.

**Table 2:** Two-part model results.

Variables	Panel A: Knowledge About Tobacco’s Ill Health Effects			Panel B: Knowledge About Specific Diseases Caused by Smoking		
	(1)	(2)	(3)	(4)	(5)	(6)
	Part 1: Probit	Part 2: GLM	Marginal effect	Part 1: Probit	Part 2: GLM	Marginal effect
Total sample (%)	37,500	7,152		37,500	7,152	
Price (Range: PhP 0.05 to 75)	0.022	−0.042^*^		0.016	−0.021	
	(0.021)	(0.018)		(0.018)	(0.017)	
Knowledge on Tobacco’s	−0.289^***^	−0.142	−1.014***			
Health Effects	(0.079)	(0.091)	(0.190)			
Knowledge x Price	−0.031	0.025				
	(0.020)	(0.017)				
Knowledge on Diseases				−0.102^***^	−0.066^**^	−0.366***
Caused by Smoking				(0.020)	(0.021)	(0.034)
Knowledge x Price				−0.009	0.001	
				(0.006)	(0.006)	
Age (Range: 15–101)	0.004^***^	0.007^***^		0.004^***^	0.007^***^	
	(0.000)	(0.001)		(0.005)	(0.001)	
*Male (Ref.)*						
Female	−1.372^***^	−0.433^***^		−1.374^***^	−0.435^***^	
	(0.019)	(0.040)		(0.019)	(0.041)	
*Wealth*						
*Quintile 1 (Ref.)*						
Quintile 2	−0.112^***^	0.023		−0.107^***^	0.027	
	(0.025)	(0.030)		(0.025)	(0.030)	
Quintile 3	−0.192^***^	0.041		−0.184^***^	0.045	
	(0.026)	(0.032)		(0.026)	(0.032)	
Quintile 4	−0.309^***^	0.098^**^		−0.299^***^	0.104^**^	
	(0.026)	(0.034)		(0.026)	(0.034)	
Quintile 5	−0.508^***^	0.120^**^		−0.494^***^	0.131^***^	
	(0.028)	(0.038)		(0.028)	(0.038)	
*Rural (Ref.)*						
Urban	0.064^***^	−0.017		0.077^***^	−0.009	
	(0.018)	(0.023)		(0.018)	(0.023)	
*2009 (Ref)*						
2015	−0.176^***^	0.010		−0.166^***^	0.014	
	(0.023)	(0.030)		(0.023)	(0.031)	
2021	−0.547^***^	0.081		−0.522^***^	0.091^*^	
	(0.037)	(0.047)		(0.037)	(0.046)	
Constant	0.246^**^	2.112^***^		0.203^***^	2.126^***^	
	(0.082)	(0.098)		(0.059)	(0.063)	

Notes: *** *p* < 0.001, ** *p* < 0.05, * *p* < 0.1 (two-tailed tests); Robust standard errors in parentheses. Probit results (Columns 1 and 4) are in predicted probabilities.

**Fig 1:**
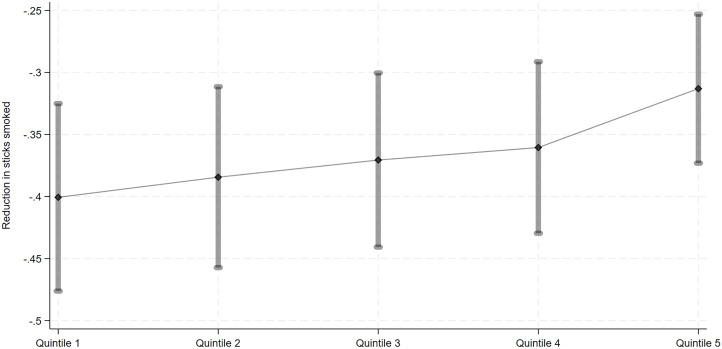
Knowledge Score and Number of Sticks Smoked by Wealth Quintile (Marginal Effect). Graph shows the positive association between knowledge scores and the reduction in sticks smoked (y-axis), with the strength of this association increasing across wealth quintiles (x-axis, from the poorest to the wealthiest groups).

As for the second outcome variable, we found knowledge about specific smoking-related diseases to be negatively associated with smoking. In particular, a unit increase in knowledge score is associated with a 0.37-unit average reduction (4.0% reduction compared to the 9.22-stick average) in sticks smoked (Table 2, column 6). When examined further by looking at wealth quintiles, we found the association between knowledge score and the number of sticks smoked to be more pronounced for individuals from the lowest wealth quintile (0.40-unit reduction in sticks smoked) compared to those in the highest quintile (0.31-unit reduction in sticks smoked). These results are shown in Fig 1. Pairwise comparison results offer support to the claim that the marginal effect of knowledge on the number of sticks smoked are significantly different between individuals in the lowest and highest quintiles (S2 Table).

## Discussion

Two main ideas stem from this paper’s results. First, knowledge about smoking’s ill health effects continues to play a significant role in people’s smoking decision, even when considering a baseline where a great majority of individuals (>90%) are aware about smoking’s health risks. Second, despite gains, significant knowledge disparities persist across wealth groups – a fact that can lie hidden when looking at population-wide improvements in the public’s understanding of the risks attributed to smoking. Consistent with other studies, health-related knowledge was observed to be higher among non-smokers than smokers, and similarly, among individuals with higher socioeconomic status [34,35]. Descriptive statistics showed that more than 9 in 10 adult Filipinos, smoker and non-smoker alike, are aware of the health risks associated with smoking, yet the extent of individuals’ awareness varied with wealth. That significant differences persisted opens issues about knowledge- or information-based public health programs and how to tailor them.

From a public policy standpoint, two relevant questions arise: (a) What type of information would elicit a behavioral change from smokers, and (b) How should persistent health disparities by socioeconomic status inform the design of public health programs? Among policies aimed to raise awareness about smoking’s health risks, cigarette pack warnings have been shown to discourage smoking and increase chances of successfully quitting [36]. Health warnings come in different types, settings, and specifications – from text to graphic warnings. Size and design specifications have been shown as important considerations for policies aimed at increasing health knowledge and cessation [9,37]. Little is known about individuals’ response to the policy given a paucity of related studies on the effect of the two laws in the Philippines – RA 9211 and RA 10643 – providing for health warnings in cigarette packs. Earlier research that explored perceptions using online and household surveys found Philippine graphic warnings to be less potent in discouraging smoking when compared to Singapore’s and Thailand’s policies where health warnings occupied a larger portion of each pack (85% of each pack in Thailand and Singapore vs. 50% in the Philippines) [38]. However, research covering different countries pointed to graphic warnings as being generally effective across multiple contexts [39].

The other challenge is bridging the wealth-based knowledge gap, especially among individuals in the lowest wealth groups. Besides size and design as important considerations in increasing effectiveness of health warnings, other factors come into play when dealing with health promotion for different population groups. Related research pointed to message engagement as the dominant factor affecting effectiveness of smoking-related health information initiatives among low socioeconomic status groups, with individuals trusting lay over expert messaging and giving value to messages they can identify with [40]. This idea calls for more relevant approaches to increase awareness about smoking’s health risks. In the Philippines, *barangay* or community health workers (CHW) are able to serve as bridge between the population and the primary health care system due to the trust they enjoy among community members [41,42]. CHWs in the Philippines are volunteers who work under the guidance of local governments, receiving support in the form of training and honoraria. Earlier work on CHWs highlights the trust and legitimacy this group enjoys, emanating not from professionalization but from good social relations established with their communities [43] In this sense, integrating health information with CHW services has the potential to effectively raise awareness and discourage smoking among individuals in the lowest wealth groups, especially more nicotine dependent smokers who are unlikely to quit smoking as a result of other types of interventions [44].

### Limitations

Several limitations should be noted when interpreting the results of this cross-sectional study. First, variables on smoking-related knowledge were based on self-reported information. Thus, we could not rule out bias that could arise from self-reported data. Second, the additive knowledge score was limited to the three smoking-related diseases that are common to all three GATS waves. Data limitations prevented us from creating a score with a longer list of smoking-related health conditions. Third, imputations and self-reported information were used to determine the price variable. This was to address potential endogeneity issues. However, as with the other variables, we could not rule out possible measurement error arising from self-reported information.

## Conclusion

Increasing knowledge about smoking’s health risks remains an important issue despite significant gains in public awareness about the dangers of direct and secondhand smoking. The introduction of various public health policies – from health warnings in cigarette packs to anti-smoking advertisements – have contributed to wider understanding of the harms of smoking. However, as this paper emphasized, knowledge-based gains remained inequitably distributed across population groups. People with lower socioeconomic status, particularly those in the lowest wealth groups who are typically the ones most affected by smoking, significantly differed in terms of smoking-related knowledge when compared to those in the top wealth groups. This issue calls for innovative approaches to health information-based programs that consider the needs and context of individuals in the lowest socioeconomic groups, with the aim of closing knowledge-based disparities.

## Supporting information

S1 Table Descriptive Statistics, ANOVA Results, and Pairwise Comparisons for Wealth Quintiles on KAP scores (Tukey HSD), 2009, 2015, and 2021. Note. SS = Sum of Squares; df = degrees of freedom; MS = Mean Square; F = F-statistic; p = p-value; CI = Confidence Interval; PWC = pairwise comparison (of quintiles).
(DOCX)

S2 Table Pairwise Comparison by Wealth Quintile (Marginal Effect of Knowledge Scores on Number of Sticks Smoked). Note. Results are based on the delta method. Unadjusted 95% confidence intervals are reported.
(DOCX)
