# Supplementary material for: Smoking knowledge and decision in an era of widespread awareness: Persistent disparities and policy implications
Source: PLoS One. 2025 Aug 4;20(8):e0329691. doi: 10.1371/journal.pone.0329691 (PMC12321053; doi:10.1371/journal.pone.0329691)
Supplement: S1 Table — Note. SS = Sum of Squares; df = degrees of freedom; MS = Mean Square; F = F-statistic; p = p-value; CI = Confidence Interval; PWC = pairwise comparison (of quintiles). (DOCX) [file pone.0329691.s001.docx]

**Table S.1.** **Descriptive Statistics, ANOVA Results, and Pairwise Comparisons for Wealth Quintiles on KAP scores (Tukey HSD), 2009, 2015, and 2021**

|  | **Panel A: Descriptive Statistics** | | | | **Panel B: ANOVA** | | | | | **Panel C: Tukey HSD** | | | | | | |
| --- | --- | --- | --- | --- | --- | --- | --- | --- | --- | --- | --- | --- | --- | --- | --- | --- |
| **Period** | **Wealth Quintile** | **Mean** | **Std. Dev.** | **Freq** | **ANOVA (SS)** | **df** | **MS** | **F** | **p-value** | **PWC** | **Contrast** | **Std. Error** | **t** | **p-value** | **Lower CI** | **Upper CI** |
| 2009 | 1 (Lowest) | 2.361 | 0.897 | 1,830 |  |  |  |  |  | 2 vs 1 | 0.127 | 0.025 | 5.170 | 0.000 | 0.060 | 0.194 |
|  | 2 | 2.488 | 0.805 | 1,978 |  |  |  |  |  | 3 vs 1 | 0.211 | 0.026 | 8.190 | 0.000 | 0.140 | 0.281 |
|  | 3 | 2.572 | 0.754 | 1,667 |  |  |  |  |  | 4 vs 1 | 0.271 | 0.025 | 10.870 | 0.000 | 0.203 | 0.339 |
|  | 4 | 2.632 | 0.717 | 1,888 |  |  |  |  |  | 5 vs 1 | 0.399 | 0.025 | 15.940 | 0.000 | 0.331 | 0.467 |
|  | 5 (Highest) | 2.760 | 0.585 | 1,844 |  |  |  |  |  | 3 vs 2 | 0.083 | 0.025 | 3.300 | 0.009 | 0.015 | 0.152 |
|  | **Total** | 2.562 | 0.771 | 9,207 |  |  |  |  |  | 4 vs 2 | 0.144 | 0.024 | 5.880 | 0.000 | 0.077 | 0.210 |
|  |  |  |  |  |  |  |  |  |  | 5 vs 2 | 0.272 | 0.025 | 11.070 | 0.000 | 0.205 | 0.339 |
|  | **Model** |  |  |  | 166.40 | 4 | 41.60 | 72.23 | <0.01 | 4 vs 3 | 0.060 | 0.026 | 2.360 | 0.126 | -0.009 | 0.130 |
|  | Residual |  |  |  | 5299.88 | 9,202 | 0.58 |  |  | 5 vs 3 | 0.189 | 0.026 | 7.350 | 0.000 | 0.119 | 0.259 |
|  | Total |  |  |  | 5466.28 | 9,206 | 0.59 |  |  | 5 vs 4 | 0.128 | 0.025 | 5.170 | 0.000 | 0.061 | 0.196 |
| 2015 | 1 (Lowest) | 2.484 | 0.846 | 2,540 |  |  |  |  |  | 2 vs 1 | 0.149 | 0.021 | 7.160 | 0.000 | 0.092 | 0.206 |
|  | 2 | 2.633 | 0.725 | 2,210 |  |  |  |  |  | 3 vs 1 | 0.164 | 0.021 | 7.740 | 0.000 | 0.106 | 0.222 |
|  | 3 | 2.648 | 0.704 | 2,056 |  |  |  |  |  | 4 vs 1 | 0.220 | 0.021 | 10.620 | 0.000 | 0.164 | 0.277 |
|  | 4 | 2.704 | 0.641 | 2,251 |  |  |  |  |  | 5 vs 1 | 0.251 | 0.021 | 12.160 | 0.000 | 0.195 | 0.307 |
|  | 5 (Highest) | 2.734 | 0.626 | 2,292 |  |  |  |  |  | 3 vs 2 | 0.015 | 0.022 | 0.700 | 0.957 | -0.045 | 0.075 |
|  | **Total** | 2.637 | 0.722 | 11,349 |  |  |  |  |  | 4 vs 2 | 0.071 | 0.021 | 3.320 | 0.008 | 0.013 | 0.130 |
|  |  |  |  |  |  |  |  |  |  | 5 vs 2 | 0.102 | 0.021 | 4.760 | 0.000 | 0.044 | 0.160 |
|  | **Model** |  |  |  | 91.87 | 4 | 22.97 | 44.77 | <0.01 | 4 vs 3 | 0.056 | 0.022 | 2.560 | 0.079 | -0.004 | 0.115 |
|  | Residual |  |  |  | 5819.55 | 11,344 | 0.51 |  |  | 5 vs 3 | 0.086 | 0.022 | 3.970 | 0.001 | 0.027 | 0.146 |
|  | Total |  |  |  | 5911.42 | 11,348 | 0.52 |  |  | 5 vs 4 | 0.031 | 0.021 | 1.440 | 0.602 | -0.027 | 0.089 |
| 2021 | 1 (Lowest) | 2.746 | 0.621 | 4,358 |  |  |  |  |  | 2 vs 1 | 0.057 | 0.013 | 4.570 | 0.000 | 0.023 | 0.092 |
|  | 2 | 2.804 | 0.535 | 2,949 |  |  |  |  |  | 3 vs 1 | 0.082 | 0.012 | 6.910 | 0.000 | 0.050 | 0.114 |
|  | 3 | 2.828 | 0.503 | 3,646 |  |  |  |  |  | 4 vs 1 | 0.094 | 0.012 | 7.990 | 0.000 | 0.062 | 0.127 |
|  | 4 | 2.841 | 0.469 | 3,671 |  |  |  |  |  | 5 vs 1 | 0.096 | 0.012 | 8.150 | 0.000 | 0.064 | 0.129 |
|  | 5 (Highest) | 2.843 | 0.477 | 3,660 |  |  |  |  |  | 3 vs 2 | 0.024 | 0.013 | 1.870 | 0.336 | -0.011 | 0.060 |
|  | **Total** | 2.810 | 0.529 | 18,284 |  |  |  |  |  | 4 vs 2 | 0.037 | 0.013 | 2.840 | 0.037 | 0.001 | 0.073 |
|  |  |  |  |  |  |  |  |  |  | 5 vs 2 | 0.039 | 0.013 | 3.000 | 0.024 | 0.003 | 0.075 |
|  | **Model** |  |  |  | 26.38 | 4 | 6.59 | 23.70 | <0.01 | 4 vs 3 | 0.013 | 0.012 | 1.020 | 0.845 | -0.021 | 0.046 |
|  | Residual |  |  |  | 5084.94 | 18,279 | 0.28 |  |  | 5 vs 3 | 0.015 | 0.012 | 1.180 | 0.762 | -0.019 | 0.048 |
|  | Total |  |  |  | 5111.31 | 18,283 | 0.28 |  |  | 5 vs 4 | 0.002 | 0.012 | 0.160 | 1.000 | -0.032 | 0.036 |

*Note.* SS = Sum of Squares; df = degrees of freedom; MS = Mean Square; F = F-statistic; p = p-value; CI = Confidence Interval; PWC = pairwise comparison (of quintiles)
