# Supplementary material for: Smoking knowledge and decision in an era of widespread awareness: Persistent disparities and policy implications
Source: PLoS One. 2025 Aug 4;20(8):e0329691. doi: 10.1371/journal.pone.0329691 (PMC12321053; doi:10.1371/journal.pone.0329691)
Supplement: S2 Table — Note. Results are based on the delta method. Unadjusted 95% confidence intervals are reported. (DOCX) [file pone.0329691.s002.docx]

**Table S.2.** **Pairwise Comparison by Wealth Quintile (Marginal Effect of Knowledge Scores on Number of Sticks Smoked)**

| **Quintile Pair** | **Contrast** | **Delta Method**  **Std. Error** | **95% CI Lower** | **95% CI Upper** |
| --- | --- | --- | --- | --- |
| 3 vs 1 | -0.2836 | 0.0801 | -0.4405 | -0.1267 |
| 2 vs 1 | -0.1621 | 0.0784 | -0.3157 | -0.0085 |
| 4 vs 1 | -0.4044 | 0.0807 | -0.5626 | -0.2461 |
| 5 vs 1 | -0.7242 | 0.0812 | -0.8833 | -0.5651 |
| 3 vs 2 | -0.1214 | 0.0814 | -0.2810 | 0.0381 |
| 4 vs 2 | -0.2423 | 0.0813 | -0.4016 | -0.0829 |
| 5 vs 2 | -0.5621 | 0.0812 | -0.7212 | -0.4030 |
| 4 vs 3 | -0.1208 | 0.0822 | -0.2819 | 0.0403 |
| 5 vs 3 | -0.4407 | 0.0813 | -0.6000 | -0.2813 |
| 5 vs 4 | -0.3198 | 0.0804 | -0.4774 | -0.1623 |

*Note.* Results are based on the delta method. Unadjusted 95% confidence intervals are reported
